# Comprehensive genomic profiling on metastatic Melanoma: results from a network screening from 7 Italian Cancer Centres

**DOI:** 10.1186/s12967-023-04776-2

**Published:** 2024-01-06

**Authors:** Matteo Pallocca, Ivan Molineris, Enrico Berrino, Benedetta Marcozzi, Martina Betti, Lauretta Levati, Stefania D’Atri, Chiara Menin, Gabriele Madonna, Paola Ghiorzo, Jenny Bulgarelli, Virgina Ferraresi, Tiziana Venesio, Monica Rodolfo, Licia Rivoltini, Luisa Lanfrancone, Paolo Antonio Ascierto, Luca Mazzarella, Pier Giuseppe Pelicci, Ruggero De Maria, Gennaro Ciliberto, Enzo Medico, Giandomenico Russo

**Affiliations:** 1 Institute of Experimental Endocrinology and Oncology, National Research Council, Naples, Italy; 2 Department of Life Science and System Biology, University of Turin, Via Accademia Albertina 13, 10123 Turin, Italy; 3 Candiolo Cancer Institute, FPO-IRCCS, Candiolo, Italy; 4 University of Turin, Turin, Italy; 5 Biostatistics, Bioinformatics and Clinical Trial Center, IRCCS Regina Elena National Cancer Institute, Rome, Italy; 6 Laboratory of Molecular Oncology, IDI-IRCCS, Rome, Italy; 7 Immunology and Oncological Molecular Diagnostics, Oncological Institute, IOV IRCCS UOC, Padua, Italy; 8 Melanoma, Cancer Immunotherapy and Development Therapeutics, Istituto Nazionale Tumori IRCCS Fondazione G. Pascale, 80131 Naples, Italy; 9 Genetics of Rare Cancers, IRCCS Ospedale Policlinico San Martino, 16132 Genoa, Italy; 10 Department of Internal Medicine and Medical Specialties, University of Genova, 16132 Genoa, Italy; 11 Immunotherapy, Cell Therapy and Biobank Unit, IRCCS Istituto Romagnolo Per lo Studio dei Tumori (IRST) “Dino Amadori”, 47014 Meldola, Italy; 12 Sarcoma and Rare Tumours Departmental Unit- IRCCS Regina Elena National Cancer Institute-Rome, Rome, Italy; 13 Unit of Translational Immunology, Department of Experimental Oncology, IRCCS Foundation National Cancer Institute, Milan, Italy; 14 Department of Experimental Oncology, European Institute of Oncology IRCCS (IEO), Milan, Italy; 15 Department of Oncology and Hemato-Oncology, University of Milan, Milan, Italy; 16 Institute of General Pathology, Catholic University “Sacro Cuore”, Rome, Italy; 17 Scientific Direction, IRCCS Regina Elena National Cancer Institute, Rome, Italy; 18 Istituto Dermopatico dell’Immacolata, IDI-IRCCS, Rome, Italy

**Keywords:** Comprehensive genomic profiling, Network trial, Alleanza Contro il Cancro, Melanoma, SKCM, Immuno-checkpoint inhibitors, Genomic biomarkers

## Abstract

**Background:**

The current therapeutic algorithm for Advanced Stage Melanoma comprises of alternating lines of Targeted and Immuno-therapy, mostly via Immune-Checkpoint blockade. While Comprehensive Genomic Profiling of solid tumours has been approved as a companion diagnostic, still no approved predictive biomarkers are available for Melanoma aside from BRAF mutations and the controversial Tumor Mutational Burden. This study presents the results of a Multi-Centre Observational Clinical Trial of Comprehensive Genomic Profiling on Target and Immuno-therapy treated advanced Melanoma.

**Methods:**

82 samples, collected from 7 Italian Cancer Centres of FFPE-archived Metastatic Melanoma and matched blood were sequenced via a custom-made 184-gene amplicon-based NGS panel. Sequencing and bioinformatics analysis was performed at a central hub. Primary analysis was carried out via the Ion Reporter framework. Secondary analysis and Machine Learning modelling comprising of uni and multivariate, COX/Lasso combination, and Random Forest, was implemented via custom R/Python scripting.

**Results:**

The genomics landscape of the ACC-mela cohort is comparable at the somatic level for Single Nucleotide Variants and INDELs aside a few gene targets. All the clinically relevant targets such as BRAF and NRAS have a comparable distribution thus suggesting the value of larger scale sequencing in melanoma. No comparability is reached at the CNV level due to biotechnological biases and cohort numerosity. Tumour Mutational Burden is slightly higher in median for Complete Responders but fails to achieve statistical significance in Kaplan–Meier survival analysis via several thresholding strategies. Mutations on PDGFRB, NOTCH3 and RET were shown to have a positive effect on Immune-checkpoint treatment Overall and Disease-Free Survival, while variants in NOTCH4 were found to be detrimental for both endpoints.

**Conclusions:**

The results presented in this study show the value and the challenge of a genomics-driven network trial. The data can be also a valuable resource as a validation cohort for Immunotherapy and Target therapy genomic biomarker research.

**Supplementary Information:**

The online version contains supplementary material available at 10.1186/s12967-023-04776-2.

## Introduction

Despite recent advancements in novel treatment and procedures, metastatic Skin Cutaneous Melanoma still has a 5-year survival of 20% and accounts for most skin cancer deaths [1]. According to the International Agency for Research on Cancer, the number of new cases diagnosed each year is increasing worldwide. The current therapeutic algorithm for stage IV melanoma comprises Targeted Therapy for patients positive to BRAF V600E mutations, followed by Immune-Checkpoint blockade as a preferential step via PD1 and/or CTLA4 inhibition [2] and chemotherapy.

Additional lines of non-BRAF targeted therapy or Immunotherapy may be assigned if the tumour Genomic Profile exhibits alterations that confer sensitivity to said treatments. While current guidelines only provide a 3-gene panel as mandatory (namely BRAF, NRAS and c-KIT), it has been shown that a larger genomics screen may inform treatment decision and predict response to Target Therapy, and partially, to Immune-checkpoint inhibitors [3–5]. Given the current suboptimal response rate of 30% and over 90% to respectively, immunotherapy and target therapy [6], is reasonable to assume that a more genomic-tailored approach would improve treatment efficacy.

We hereby present the results of an observational trial on 82 stage III-IV melanoma patients treated with Targeted and Immuno-therapy. An in-house developed panel for Comprehensive Genomic Profiling on tumour tissue was performed and provided insights on how the mutational and genomic landscape affects treatment response and survival. Furthermore, we develop a series of Survival-predicting Machine Learning models on said data and show their efficacy and limitations on CGP mutational profiles.

## Methods

### Retrospective patient enrolment and sample collection

Patients with unresectable stage IIIC or stage IV melanoma of the skin were retrospectively enrolled by the 7 institutes involved in the study, when satisfied the following criteria: (i) pre-determined BRAF mutation status; (ii) treatment with BRAFi + MEK + or with anti-PD-1 antibodies in first line; (iii) available FFPE tissue representative of the lesion before the start of therapy and (iv) available medical history information is available. Therapy response was assessed via RECIST 1.1 criteria for BRAF-MEK inhibitors and via IrRECIST for anti-PD-1 treatment. All patients provided written informed consent to the study procedures. Haematoxylin and eosin staining for the tumor cellularity identification together with the DNA extraction with the GeneRead DNA FFPE Kit (QIAGEN) and the DNA quantification with a fluorometric assay (Qubit, Thermo Fisher Scientific) were performed in each institute, each following the Standardized Operative Procedures (Additional file 1: Methods). DNA Sequencing was centralized in a single cancer center.

### Sequencing

All samples were sequenced via the custom-designed amplicon panel, comprising of 8320 targets for a total 0.8 Mb target. The average sequencing depth per sample was 769 ± 426, with 8 samples per run on a Thermo Fisher S5 sequencer. The ACC panel 1 list and design have been previously described [7], its content representing all actionable genes and oncogenic drivers known at the time of its design. Briefly, a total of 40 ng of DNA was used to prepare libraries using the Ion AmpliSeq Library kit 2.0 (Thermo Fisher Scientific) following the manufacturer’s instructions. Each library was indexed with both Ion Xpress Barcode and Ion P1 Adapter (Thermo Fisher Scientific), quantified with the Ion Library TaqMan Quantitation Kit (Thermo Fisher Scientific) a diluted to a loading concentration of 50 pM. Ion 540 Chip was loaded with 8 samples (4 tumours and 4 normal matched specimens) on the Ion Chef System (Thermo Fisher Scientific) by using the Ion 540 Kit-Chef. Sequencing was performed on the Ion GeneStudio S5 Plus System instrument (Thermo Fisher Scientific) for an expected mean read depth of 700 ×. The obtained average sequencing depth per sample was 769 ± 426. Each institute performed BRAF mutant detection validation, through either qPCR, sanger sequencing, pyrosequencing analysis, or a combination of the three.

### Bioinformatics analysis

All raw NGS data were analysed via the Thermo Fisher Ion Reporter version 5.10, using the workflow ACC_melanoma-v2 described at https://acc-bioinfo.gitlab.io. VCF were annotated using ANNOVAR. MAF files from VCFs were obtained by maftools. Copy Number alterations were calculated with a baseline across all male ACC-mela samples via the Ion Reporter cloud framework. Given the discrete numerosity of the baseline, only stringent event such as strong deletions (ploidy = 0) and large amplification (ploidy > 4) were considered. ICI Responders were classified by grouping the CR and PR response groups, while PD and SD were considered non-responders. The Tumour Mutational Burden (TMB) was calculated by dividing the total number of mutations per sample by the total number of bases sequenced. Survival curves and Random Survival Forest were carried out via the *survival*, *survminer*, and *randomForestSRC* packages, respectively. Feature selection and interpretation were conducted through the Variable IMportance Perdictor (VIMP) method, by employing the same package used for training the model, randomForestSRC; this method adopts a prediction-based approach by measuring prediction error attributable to the variable. Statistical modeling and data visualization were carried out via *ggplot*, *Complex Heatmap* [8] and *glmnet*. All custom scripts are available at https://gitlab.com/bioinfo-ire-release/acc-melanoma.

### Public data sources and clinical endpoints

All Data from the Immunogenomic and Target therapy studies was fetched via the cBioPortal, Data from two WES Immunogenomic studies (UCLA Cell 2016 and MSK NEJM 2014) and one Target therapy study (MSK Clin Cancer Res 2021) [9]. Somatic variants were selected via the VAF > 0.05 and deleterious variations filter. The Fold Change (FC) between our cohort and public cohorts was calculated by computing the difference in the mutation penetrance, adjusted by the mean penetrance of each mutation in the public cohorts (mean percentage change).

Three clinical endpoints have been considered: Overall Survival (OS), Progression Free Survival (PFS) and Response to Immuno-Checkpoints Inhibitors (ICI Response). However, since targeted data from MSK did not provide the continuous annotation for PFS (months to recurrence), results for this clinical endpoint have only been validated on the WES datasets. Moreover, ICI response annotation was not enriched with a time variable, so it was not considered for the Lasso-Cox and VIMP feature selection step.

## Results

### Genomic and actionability landscaping of melanoma via comprehensive genomic profiling

**Fig. 1:**
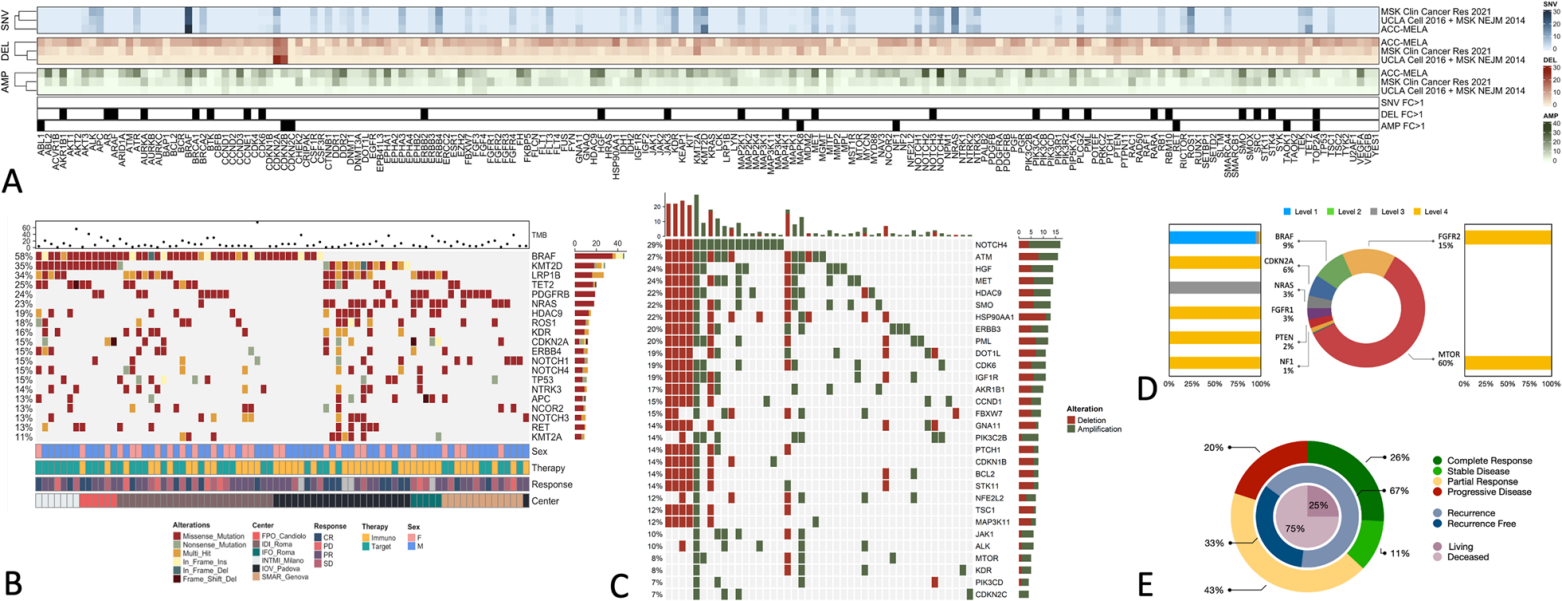
**A** Occurrence Heatmap representing differences among genomic events in the ACC-mela cohorts and other large melanoma casuistries. **B** Oncoplot representing major clinical annotations and genomic alterations available in the ACC-mela cohort. **C** Most represented Copy Number Alterations in the cohort at the gene-level. **D** Donut plot with the distribution of OncoKB actionability levels in the cohort. **E** Donut plot representing the Immune-Checkpoint inhibitor landscape of Response, Overall Survival and Recurrence

The multi-centre cohort of ACC-mela comprises of 82 stage III-IV patients, collected across all centres, with a numerosity of 46 (56%) of Immune-Checkpoint treated and 36 (44%) of patients treated with Target Therapy, namely BRAF/MEK inhibitors (Table 1). When comparing the genomic landscape of the cohort to larger casuistries (Fig. 1A), a few genes are over-represented in the mutational ratio of ACC-mela, namely MAPK1, and DDR2 (Fold Change 0.59 and 0.51, respectively). This asymmetry might be attributed to the difference in detection resolution for Targeted Sequencing (down to 5% of VAF) when compared to Whole Exome Sequencing (from 10 to 20% VAF) and in the smaller numerosity of the ACC-mela cohort (Fig. 1B). Of note, clinically relevant targets such as BRAF and NRAS have comparable incidences (53–58% for BRAF and 23–28% for NRAS, respectively) thus suggesting the value of larger scale sequencing in the melanoma context.

**Table 1:** Patient characteristics

Variable	Therapy received	
	Immuno, N = 46^1^	Target, N = 36^1^
Age at therapy start		
N	46	36
Median (IQR)	67 (60, 73)	52 (48, 67)
Range	37, 92	26, 80
Survival time (months)		
N	41	36
Median (IQR)	17 (6, 26)	20 (12, 29)
Range	0, 51	3, 51
Sex		
Female	17 (37%)	12 (33%)
Male	29 (63%)	24 (67%)
Response		
CR	9 (21%)	10 (28%)
PD	7 (17%)	2 (5.6%)
PR	15 (36%)	18 (50%)
SD	11 (26%)	6 (17%)
Status		
Alive	32 (71%)	23 (64%)
Death	13 (29%)	13 (36%)
Cause of death		
Alive or death by other causes	36 (78%)	23 (64%)
Death by melanome	10 (22%)	13 (36%)
Stage at therapy start		
IIIC	2 (4.3%)	6 (17%)
IV	1 (2.2%)	1 (2.8%)
IV_M1a	12 (26%)	6 (17%)
IV_M1b	12 (26%)	4 (11%)
IV_M1c	19 (41%)	18 (50%)
IV_M1d	0 (0%)	1 (2.8%)

^1^n (%)

Copy Number Variations frequencies for the large genomics events considered in the panel analysis (deep deletions and large amplifications), showed a Fold Change variation > 1 for deletions and amplifications on, respectively, 13.1% and 4.3% of all the genes considered in the ACC panel (Fig. 1A). We consider this dramatic imbalance be due to the strong difference in the approaches, from Amplicon-based CNV calling (Fig. 1C) to GISTIC segmentation over SNP array results (TCGA). It is feasible to assume that given a larger amplicon-based casuistry the CNV calling algorithm would stabilize towards more comparable results.

Finally, to rank the number of patients with a high level of drug actionability given the ACC-mela CGP, we annotated all somatic mutations and CNVs through the OncoKB actionability scale (Fig. 1D). Considering the BRAF mutations that represent a major event bi-partitioning the overall dataset, a 14.3% of the patient exhibited a high level of actionability status (levels A / 1) and a 13.7% of patients harboured a variant that could be targeted via a putative repurposing (levels B-C / 2–3).

### BRAF status validation

As for the consistency of the NGS approach, we detected 46/83 patients with a BRAF, codon 600 mutation. Considering the BRAF mutated ones, 45/46 had been diagnosed as BRAF mutated by the routine molecular test, with a 98% concordance. The single discordant sample carried a BRAF p.V600K, identified only by our NGS method, with a VAF of 7% and centrally confirmed with a qPCR assay (Easy BRAF, Diatech Pharmacogenetics). For this patient, the molecular diagnosis was performed by Sanger sequencing, and we can speculate that this discordance could be associated to the limit of detection of the Sanger method. All BRAF, codon 600 WT patients were confirmed by our NGS panel, for an overall concordance of 82/83 patients (99%).

### Immune-checkpoint response biomarker prediction

CGP of solid tumours has been shown to provide solid indications towards not only targeted but also Immuno-therapy, mostly via Immuno-Checkpoint inhibitors [10]. To date, only a few DNA-based events are recognized to be clinically relevant such as the Tumor Mutational Burden (TMB), defined as the amount of somatic variation across the sampled genome. TMB was proposed to be a valuable biomarker in many scenarios [11, 12] even if its static thresholding and predictive power among tumour types have been strongly criticized [13, 14] and its application from targeted panels instead of Whole Exomes still represents a technical challenge [15].

**Fig. 2:**
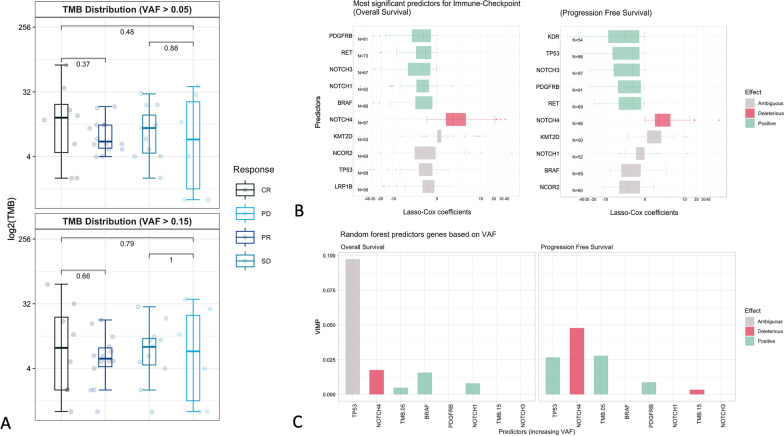
**A** TMB distribution [log_2_(TMB)] among ICI Response groups for different VAF filters. **B** Genes predicting for Overall Survival and Progression Free Survival in ICI-treated patients from the Cox Lasso Models. **C** Features predicting Disease Free Survival and Overall Survival. The VIMP value represents the weight of each feature, while the effect is represented by a color-coding which summarises the qualitative information of variables marginal effect represented in Additional file 1: Figure S2

We designed an automated process to compute TMB biomarker accuracy in predicting ICI responders when varying its minimal Variant Allele Frequency, mutation type, and TMB high/low thresholding. When comparing ACC-mela TMB stratification power to other large melanoma casuistries [16, 17], in our cohort TMB fails to reach statistical significance at 5% and 15% VAF filtering, respectively (p-value 0.38 and 0.68, respectively, Additional file 1: Figure S1). Nonetheless, median values of TMB are higher in Complete Responders than Progressive Diseases but with a highly variable distribution, with median TMB values of 12.5 in CR and 8 in PD, at 5% VAF filtering (p-value 0.48 and 0.79, respectively, Fig. 2A).

Next, we sought to consider the effect of single mutations to the Overall Survival and Progression Free Survival during ICI treatment (Fig. 1E). All genomic events were ranked via a COX uni/multivariate model of mutational status influence on Overall and Progression Free Survival. LASSO Cox regression was used for variable selection and shrinkage in Cox’s proportional hazards model. The method is a variation of the ‘lasso’ proposal designed for the linear regression context [18]. We obtained 100 plausible models and selected the top 10 most frequently significantly associated with both survival time (Fig. 2B). For both Overall and Progression Free Survival, mutations in NOTCH4 had a negative impact on ICI associated survival. In Overall Survival, patients harbouring somatic variations in PGFRB, RET, NOTCH3, NOTCH1 and BRAF showed to have a better prognosis. Other genes, such as KMT2D, NCOR2, TP53 and LRP1B, show a coefficient distribution spanning across negative and positive values, resulting in a prediction power shared among gene clusters. In Progression Free Survival, mutations KDR, TP53, NOTCH3, PDGFRB and RET showed a good impact on ICI associated survival while genes such as KMT2D, NOTCH1, BRAF and NCOR2 showed a predictive power shared among gene clusters. We sought to test whether a more complex model could be built on these data, but the cardinality of the dataset could not allow an internal validation of the model; therefore, we performed external validation on public WES and targeted data for both OS (Living, Deceased) and PFS (Disease Free, Relapse) prediction, along with ICI Response (Response, no Response). Predictive features selected by COX-Lasso (Fig. 2A) were considered for training along with TMB levels at VAF > 0.05 and VAF > 0.15 (Fig. 2B). To better describe gene profiles, we used average VAF values for each gene rather than their mutational status. Additional features, such as VAF mode and genes co-occurrences were considered but discarded for being extremely unbalanced. Moreover, CNVs and DELs were also excluded for the weak reliability that emerged in Fig. 1A.

Random Forest modeling showed an accuracy of 59.3% on ICI response (Additional file 1: Figure S2A), while for OS and PFS the accuracy did not exceed 50% for both WES and MSK targeted data (Additional file 1: Figure S2B, C). Interestingly, most genes were confirmed to be relevant for outcome prediction also by the VIMP method (Fig. 2C); the most robust association was found for mutations on the NOTCH4 gene, for which the negative correlation with prognosis (OS and PFS) has been found to be proportional to the VAF of the mutation itself. As previously noted in Fig. 2A for ICI response, the TMB with a VAF cut-off at 0.05 was found to be positively associated also with OS and PFS. Taken together, all these results confirm the possibility to derive and validate complex biomarkers from CGP profiles of advanced melanoma.

## Conclusions

Modern Precision Oncology is currently empowered by Comprehensive Genomic Profiling of solid tumours, which can inform physicians of predictive and prognostic genomic biomarkers and lay a strong foundation for present and future drug repurposing [19]. This revolution has brought to the first tumour-agnostic approvals for CGP [20] in Europe and the United States, but most genomic-based biomarkers still exhibit a sub-optimal accuracy, failing to optimize treatment strategies and costs for public and private health systems [21]. Interestingly, recent evidence shed light on how the tumour microenvironment influences clinical trajectories with profound characterization down to the single-cell transcriptome [10, 22, 23] Nonetheless the critical importance from the mechanistic point of view, the translational impact of these studies is still far from clinical applications and the routinely applied genomics companion diagnostics.

The current challenge lies into translating said NGS panel approvals into new evidence, by validating novel genomic targets into real-world data casuistries. This manuscript presents an additional piece of evidence in this direction: a retrospective multi-centre CGP cohort of advanced stage melanoma. The underlying biotechnology presented challenges, but amplicon-based sequencing is more representative of thousands of routinary samples currently sequenced throughout Europe, thus closer to Real-World Evidence validation.

## Supplementary Information

**Additional file 1.**. Supplementary Information.

## Data Availability

All data presented in this study and code (R software v4.1.0) used for bioinformatic analyses and figures have been uploaded in the online repository available at https://gitlab.com/bioinfo-ire-release/acc-melanoma/.

## Abbreviations

FFPE: Formalin-fixed paraffin-embedded
NGS: Next generation sequencing
ACC: Alliance against cancer
CNV: Copy number variation
ICI: Immune checkpoint inhibitor
SKCM: Skin cutaneous melanoma
CGP: Comprehensive genomic profiling
MAF: Mutation Annotation Format
VCF: Variant call format
CR: Complete response
PR: Partial response
SD: Stable disease
PD: Progressive disease
VAF: Variant allele frequency
GISTIC: Genomic identification of significant targets in cancer
SNP: Single nucleotide polymorphism
TCGA: The cancer genome atlas
TMB: Tumor mutational Burden
WES: Whole exome sequencing
OS: Overall survival
DFS: Disease-free survival
DELs: Deletions
VIMP: Variable importance
