# Correction to: Comprehensive genomic profiling on metastatic Melanoma: results from a network screening from 7 Italian Cancer Centres

**DOI:** 10.1186/s12967-024-05717-3

**Published:** 2024-10-22

**Authors:** Matteo Pallocca, Ivan Molineris, Enrico Berrino, Benedetta Marcozzi, Martina Betti, Lauretta Levati, Stefania D’Atri, Chiara Menin, Gabriele Madonna, Paola Ghiorzo, Jenny Bulgarelli, Virgina Ferraresi, Tiziana Venesio, Monica Rodolfo, Licia Rivoltini, Luisa Lanfrancone, Paolo Antonio Ascierto, Luca Mazzarella, Pier Giuseppe Pelicci, Ruggero De Maria, Gennaro Ciliberto, Enzo Medico, Giandomenico Russo

**Affiliations:** 1 Institute of Experimental Endocrinology and Oncology, National Research Council, Naples, Italy; 2 Department of Life Science and System Biology, University of Turin, Via Accademia Albertina 13, 10123 Naples, Italy; 3 Candiolo Cancer Institute, FPO-IRCCS, Candiolo, Italy; 4 University of Turin, Turin, Italy; 5 Biostatistics, Bioinformatics and Clinical Trial Center, IRCCS Regina Elena National Cancer Institute, Rome, Italy; 6 Laboratory of Molecular Oncology, IDI-IRCCS, Rome, Italy; 7 Immunology and Oncological Molecular Diagnostics, Oncological Institute, IOV IRCCS UOC, Padua, Italy; 8 Melanoma, Cancer Immunotherapy and Development Therapeutics, Istituto Nazionale Tumori IRCCS Fondazione G. Pascale, 80131 Naples, Italy; 9 Genetics of Rare Cancers, IRCCS Ospedale Policlinico San Martino, 16132 Genoa, Italy; 10 Department of Internal Medicine and Medical Specialties, University of Genova, 16132 Genoa, Italy; 11 Immunotherapy, Cell Therapy and Biobank Unit, IRCCS Istituto Romagnolo Per lo Studio dei Tumori (IRST) “Dino Amadori”, 47014 Meldola, Italy; 12 Sarcoma and Rare Tumours Departmental Unit- IRCCS Regina Elena National Cancer Institute-Rome, Rome, Italy; 13 Unit of Translational Immunology, Department of Experimental Oncology, IRCCS Foundation National Cancer Institute, Milan, Italy; 14 Department of Experimental Oncology, European Institute of Oncology IRCCS (IEO), Milan, Italy; 15 Department of Oncology and Hemato-Oncology, University of Milan, Milan, Italy; 16 Institute of General Pathology, Catholic University “Sacro Cuore”, Rome, Italy; 17 Scientific Direction, IRCCS Regina Elena National Cancer Institute, Rome, Italy; 18 Istituto Dermopatico dell’Immacolata, IDI-IRCCS, Rome, Italy

Following publication of the original article [1], we have been notified that affiliations of authors were published incorrectly.

It is now:

Matteo Pallocca1*†, Ivan Molineris2,3†, Enrico Berrino3,4†, Benedetta Marcozzi1, Martina Betti1, Lauretta Levati5,Stefania D’Atri5, Chiara Menin6, Gabriele Madonna7, Paola Ghiorzo8,9, Jenny Bulgarelli10, Virgina Ferraresi11,Tiziana Venesio3, Monica Rodolfo12, Licia Rivoltini12, Luisa Lanfrancone13, Paolo Antonio Ascierto6,Luca Mazzarella15, Pier Giuseppe Pelicci13,14, Ruggero De Maria15, Gennaro Ciliberto16, Enzo Medico3† and Giandomenico Russo17†

1. Biostatistics, Bioinformatics and Clinical Trial Center, IRCCS Regina Elena National Cancer Institute, Rome, Italy.

2. Department of Life Science and System Biology, University of Turin, Via Accademia Albertina 13, 10123 Turin, Italy.

3. University of Turin at Candiolo Cancer Institute, Turin, Italy.

4. Candiolo Cancer Institute, FPO-IRCCS, Candiolo, Italy.

5. Laboratory of Molecular Oncology, IDI-IRCCS, Rome, Italy.

6 Immunology and Oncological Molecular Diagnostics, Oncological Institute, IOV IRCCS UOC, Padua, Italy.

7. Melanoma, Cancer Immunotherapy and Development Therapeutics, Istituto Nazionale Tumori IRCCS, Fondazione G. Pascale, 80131 Naples, Italy.

8. Genetics of Rare Cancers, IRCCS Ospedale Policlinico San Martino, 16132 Genoa, Italy.

9. Department of Internal Medicine and Medical Specialties, University of Genova, 16132 Genoa, Italy.

10. Immunotherapy, Cell Therapy and Biobank Unit, IRCCS Istituto Romagnolo Per lo Studio dei Tumori (IRST) “Dino Amadori”, 47014 Meldola, Italy.

11. Sarcoma and Rare Tumours Departmental Unit- IRCCS Regina Elena National Cancer Institute-Rome, Rome, Italy.

12. Unit of Translational Immunology, Department of Experimental Oncology, IRCCS Foundation National Cancer Institute, Milan, Italy.

13. Department of Experimental Oncology, European Institute of Oncology IRCCS (IEO), Milan, Italy.

14. Department of Oncology and Hemato-Oncology, University of Milan, Milan, Italy.

15. Institute of General Pathology, Catholic University “Sacro Cuore”, Rome, Italy.

16. Scientifc Direction, IRCCS Regina Elena National Cancer Institute, Rome, Italy.

17. Istituto Dermopatico dell’Immacolata, IDI-IRCCS, Rome, Italy.

It should be:

Matteo Pallocca1*†, Ivan Molineris2,3†, Enrico Berrino3,4†, Benedetta Marcozzi 5, Martina Betti 5, Lauretta Levati 6, Stefania D’Atri 6, Chiara Menin 7, Gabriele Madonna 8, Paola Ghiorzo 9,10, Jenny Bulgarelli 11, Virgina Ferraresi 12, Tiziana Venesio 3, Monica Rodolfo 13, Licia Rivoltini 13, Luisa Lanfrancone 14, Paolo Antonio Ascierto 8, Luca Mazzarella 14, Pier Giuseppe Pelicci 14,15, Ruggero De Maria 16, Gennaro Ciliberto 17, Enzo Medico 3†, Giandomenico Russo 18†

1. Institute of Experimental Endocrinology and Oncology, National Research Council, Naples, Italy.

2. Department of Life Science and System Biology, University of Turin, Via Accademia Albertina 13, 10123, Turin, Italy.

3. Candiolo Cancer Institute, FPO-IRCCS, Candiolo, Italy.

4. University of Turin, Turin, Italy.

5. Biostatistics, Bioinformatics and Clinical Trial Center, IRCCS Regina Elena National Cancer Institute, Rome, Italy.

6. Laboratory of Molecular Oncology, IDI-IRCCS, Rome, Italy.

7. Immunology and Oncological Molecular Diagnostics, Oncological Institute, IOV IRCCS UOC, Padua, Italy.

8. Melanoma, Cancer Immunotherapy and Development Therapeutics, Istituto Nazionale Tumori IRCCS Fondazione G. Pascale, 80,131, Naples, Italy.

9. Genetics of Rare Cancers, IRCCS Ospedale Policlinico San Martino, 16,132, Genoa, Italy.

10. Department of Internal Medicine and Medical Specialties, Univers.ity of Genova, 16,132, Genoa, Italy.

11. Immunotherapy, Cell Therapy and Biobank Unit, IRCCS Istituto Romagnolo Per lo Studio dei Tumori (IRST) “Dino Amadori”, 47,014, Meldola, Italy.

12. Sarcoma and Rare Tumours Departmental Unit- IRCCS Regina Elena National Cancer Institute-Rome, Rome, Italy.

13. Unit of Translational Immunology, Department of Experimental Oncology, IRCCS Foundation National Cancer Institute, Milan, Italy.

14. Department of Experimental Oncology, European Institute of Oncology IRCCS (IEO), Milan, Italy.

15. Department of Oncology and Hemato-Oncology, University of Milan, Milan, Italy.

16. Institute of General Pathology, Catholic University “Sacro Cuore”, Rome, Italy.

17. Scientific Direction, IRCCS Regina Elena National Cancer Institute, Rome, Italy.

18. Istituto Dermopatico dell’Immacolata, IDI-IRCCS, Rome, Italy.

The original article was updated.
