# Supplementary material for: Comprehensive genomic profiling on metastatic Melanoma: results from a network screening from 7 Italian Cancer Centres
Source: J Transl Med. 2024 Jan 6;22:29. doi: 10.1186/s12967-023-04776-2 (PMC10770968; doi:10.1186/s12967-023-04776-2)

# **Supplementary Material**

**Methods**

To standardize the DNA purification, we drafted the Standard Operative Procedures. First, we shared to all the institute the ACC Melanoma Sequencing workflow, to show the steps for the FFPE management. We indicated 2 protocols for DNA extraction from blood (QIAamp DNA Blood Kits, cat. No. 51104, Qiagen or Maxwell RSC Blood DNA Kit, cat. No. AS1400, Promega).

As for FFPE DNA extraction, Mag Core^®^ Genomic DNA FFPE kit, cat.No 05-MGF-03 (MagCore), Maxwell RSC DNA FFPE Kit, cat.No AS1450 (Promega), and QIAamp DNA FFPE Tissue Kit, cat.No 56404 (Qiagen) were chosen, and reagents were sent to each institute.

Since different kits were used, we reasoned to standardize the DNA input for NGS by unfirming the quality controls before DNA sequencing, analyzing only those sample with good absorbance ratios (R230 and R280 => 1.8) and with a low level of DNA fragmentation, calculated with a qPCR-based method.

**Figures**

Figure S1: Kaplan-Meier curves of TMB high and low with different VAF thresholds (VAF = 5 and VAF= 15). Our cohort (ACC-MELA) is compared to three public validation cohorts, two WES (MSK 2014 and UCLA 2016) and one targeted (MSK 2021).


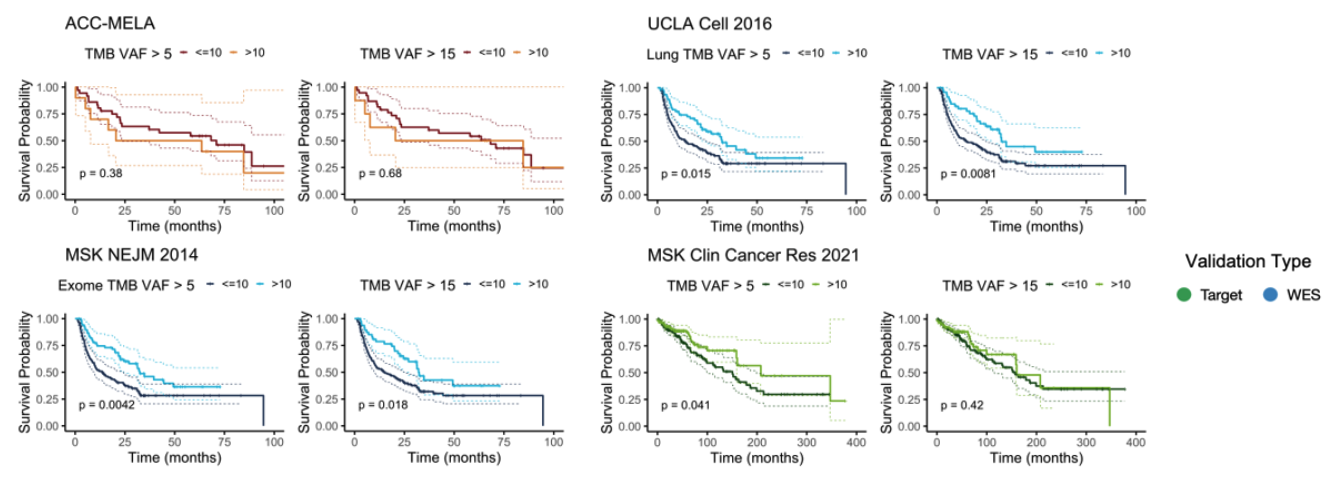


Figure S2: A) Marginal effect of predictive variables on the prediction of ICI target and the respective confusion matrix. The curves indicate the effect of increasing VAF of the mutation of each gene on the probability of negative response to treatment. B) Marginal effect of predictive variables on the prediction of PFS target and the respective confusion matrix. The curves indicate the effect of increasing VAF of the mutation of each gene on the probability of recurrence. C) Marginal effect of predictive variables on the prediction of OS target and the respective confusion matrix. The curves indicate the effect of increasing VAF of the mutation of each gene on the probability of death.


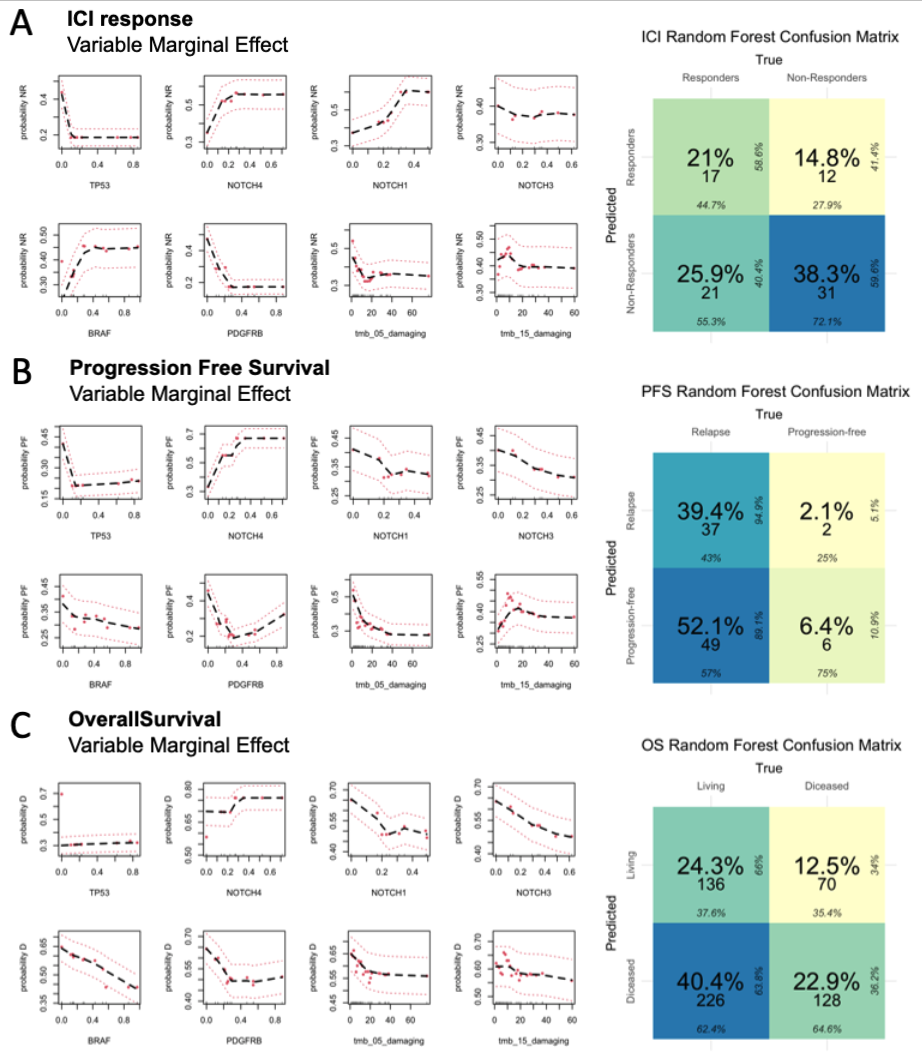

Supplement: Supplementary file 1 — Additional file 1.. Supplementary Information. [file 12967_2023_4776_MOESM1_ESM.docx]
